# A proprietary blend of quail egg for the attenuation of nasal provocation with a standardized allergenic challenge: a randomized, double-blind, placebo-controlled study

**DOI:** 10.1002/fsn3.147

**Published:** 2014-07-20

**Authors:** Annie-Claude Benichou, Marion Armanet, Anthony Bussière, Nathalie Chevreau, Jean-Michel Cardot, Jan Tétard

**Affiliations:** 1 Stragen Services SASLyon, France; 2 Stragen Pharma SAGeneva, Switzerland; 3 Chevreau Consulting LLCSalt Lake City, Utah; 4 Université d'AuvergneClermont-Ferrand, France

**Keywords:** Airborne allergen, allergic rhinitis, dietary supplement, quail egg, randomized double-blind clinical study, SniZtop

## Abstract

Occasional rhinitis symptoms caused by exposure to pollution or allergens is a growing concern. Based first on empirical observation of a lesser occurrence of allergies in quail farmers and then scientific works on ovomucoids properties, we developed a dietary supplement for the relief of such occasional rhinitis symptoms. The objective of the study was to determine whether one acute oral dose of the study product attenuates nasal provocation and other allergy-related symptoms after exposure to a standardized allergenic challenge as compared to placebo. Healthy subjects were recruited to participate in a randomized, double-blind, two-arm crossover, placebo-controlled, clinical trial. One acute dose of either the active study product (proprietary blend of quail egg) or placebo was given concomitantly to the standardized allergenic challenge. The primary endpoint was peak nasal inspiratory flow (PNIF) measurement and the secondary endpoints were subjects' perceived feelings of well-being based on Visual Analog Scale (VAS) scores for allergy-related symptoms, as well as immunoglobulin E count. Forty-three healthy subjects were enrolled and evaluable in a per protocol analysis. A gradual increase in PNIF from nadir up to Time 120 reflected the normal, gradual recovery from nasal obstruction induced by allergenic challenge for both the active and the placebo groups. At all postchallenge time points, the active group had higher PNIF values compared to the placebo group, indicating that the active product was associated with fewer symptoms and reduced intensity of these symptoms. The active product resulted also in statistically significant improvements of most of the subjects' perceived feelings of well-being based on VAS scores. No adverse events occurred during the study. In conclusion, the dietary supplement consisting of proprietary blend made of quail eggs provides fast and efficient relief of allergic rhinitis symptoms caused by the most common outdoor and indoor allergens, without adverse events.

## Introduction

Maintaining respiratory health is one of the big challenges that we face today with ongoing exposure to industrial and automobile exhaust pollution, cigarette smoke, and indoor and outdoor allergens. Groups that are particularly vulnerable are those who develop hypersensitivity to plant- or animal-based allergens such as pollens from trees, grass and weeds, pet hair and danders, and house mites (Ring et al. 2012; Asthma and Allergy Foundation of America 2010). In these individuals, exposure induces transient inflammation of the nasal airway causing various degrees of host discomfort. The symptoms are characterized by episodic nasal obstruction, rhinorrhea, sneezing, and/or itching with or without ocular involvement (Bousquet et al. 2008). These allergic responses temporarily decrease quality of life by reducing their ability to participate in activities and often initiate use of over the counter medications.

Allergic rhinitis symptoms mainly result from an immunoglobulin E (IgE)-mediated temporary immune response against environmental triggers. In individuals with a sensitized immune system, allergen-specific IgE antibodies produced in response to environmental allergens are bound to mast cells and basophil membranes (Bousquet et al. 2008). Subsequent exposure to the same allergens causes cross-linking of bound IgE by those allergens which activates mast cells and basophils, and leads to cellular degranulation and the transient release of proinflammatory mediators including histamine. This cascading pathway is responsible for immediate symptoms which can begin within a few minutes of exposure to the allergen (Calderon et al. 2007).

In the early 1970s, a French general practitioner noticed that farmers who raised quails (*Coturnix coturnix*) presented fewer allergy symptoms than the general population in the same area. He gave raw quail eggs to his allergic patients, including both adults and children, and observed a reduction in their symptoms (Truffier 1978). This finding was subsequently investigated in several human clinical trials carried out by a larger group of physicians under the direction of a highly respected French allergist physician Dr. G. Bruttmann. In these studies, subjects suffering from outdoor and indoor allergens were given quail egg powder tablets or placebo. The results of these studies indicated that consumption of quail egg powder led to relief of subjects' symptoms with good tolerability of the administered product (Bruttmann 1995). The composition of quail eggs has been described by Prelipcean (Teuşan) et al. (2012) as containing about 68% of water, 12% of proteins including ovomucoids, 10% of fats, 8% of minerals, and 2% of carbohydrates.

In vitro testing was then performed to identify the main bioactives and corresponding mechanism of action. These studies suggested that protein fractions contained in the quail egg, including ovomucoids and ovoinhibitors, act as serine protease inhibitors (Feeney et al. 1969; Takahashi et al. 1994; Vergnaud and Bruttmann 2007). Certain outdoor and indoor antigens such as pollen, mold, animal dander, and house dust mites contain protease enzymes. When they are inhaled and come into direct contact with the nasal cavity endothelium, these protease enzymes can injure tissues and induce a transient IgE-mediated allergic inflammatory response (Widmer and Whittaker 2000). By inhibiting proteases, quail egg bioactives help attenuate the episodic symptoms of an allergic reaction.

Furthermore, toxicological studies including acute and repeated oral administration on rats as well as in vitro studies demonstrated good tolerability of the product containing quail egg homogenate without mutagenic or genotoxic effects (Bruttmann 1995). Therefore, the goal of this study was to determine whether a proprietary quail egg-based dietary supplement would reduce or alleviate the physical symptoms acutely experienced by healthy subjects upon exposure to a cocktail of commonly encountered outdoor and indoor allergens.

## Materials and Methods

This study was conducted in accordance with the Declaration of Helsinki and the note for guidance on good clinical practice (ICH-GCP). The trial was approved by the IntegReview Ethical Review Board. The study was carried out from 20 June 2013 to 26 October 2013 at a clinical center in North America (California).

### Subjects

Study subjects were recruited through online or database recruitment methodologies. Subjects were enrolled in the trial if they fulfilled the following criteria: healthy volunteers between 18 and 60 years of age; occasionally experiencing allergic rhinitis symptoms that did not require daily management with antiallergic medicinal products; a baseline peak nasal inspiratory flow (PNIF) values greater than 100 L/min (average of three readings) and with a drop in PNIF >30 L/min postallergen challenge; judged by the investigator to be in general good health on the basis of their medical history. The inclusion criteria concerning baseline PNIF value and drop in PNIF postchallenge were set in order to avoid people with an already low PNIF at baseline due to concurrent disease and people who do not develop symptoms following an allergenic challenge. The minimum value of 100 L/min at baseline was defined in accordance with the results of Ottaviano et al. (2006) and the magnitude of the drop in PNIF was based on the work of Scadding et al. (2012). Main exclusion criteria included chronic diseases, cardiac disorders, respiratory diseases or airway disorders, chronic use of antihistamines (more than three times a week), and known sensitivities to the ingredients in the product. All subjects signed an informed consent form.

### Study design

This study was a randomized, double-blind, two-arm crossover, placebo-controlled clinical trial. Subjects either received one oral dose of a proprietary quail egg-based dietary supplement (PBQE) or an identical looking placebo immediately after administration of the allergen challenge. The primary variable was the PNIF as an indicator of nasal congestion. The secondary variables were the rating of nasal and ocular symptoms using a 100-mm Visual Analog Scale (VAS) and blood total IgE.

**Figure 1:**
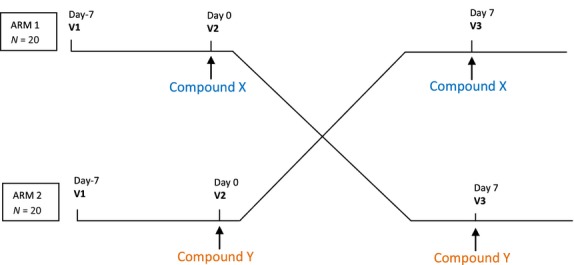
Study flowchart, crossover design trial.

The study consisted of three visits each separated by at least 7 days: Visit 1 (Screening Visit) at Day-7, Visit 2 (TEST 1) at Day 0, and Visit 3 (TEST 2 – End of Study Visit) at Day 7 (Fig.1).

A 1-week washout of any antihistamine and/or antiallergy products was required for all subjects between visits.

Visit 1 included the recording of demographic data, medical history, prior and concomitant medications, as well as physical examination, vital signs, anthropometric measures, antigen exposure protocol, urine collection for female subjects (in order to run pregnancy tests), preexposure and then multiple times postchallenge PNIF measurements over 1-h, self-assessment of subjects' symptoms by scoring the VAS at the same time points, that is, preallergen exposure, and Time = 10 (10 min postallergen exposure), Time = 20, Time = 30, Time = 45, and Time = 60.

For Visits 2 and 3, subjects arrived at the clinic having fasted for a minimum of 6 h. Visits 2 and 3 included a recording of intercurrent medical history and concomitant medication history, vital signs assessment, antigen exposure protocol, study product dispensing, PNIF measured preexposure and then multiple times postchallenge over 2 h (Time = 0, Time = 15, Time = 30, Time = 60, Time = 90, and Time = 120 min), as well as subject's self-assessment of their symptoms by scoring the VAS at the same time points. Laboratory assessments conducted at Visit 2 included serum total IgE, complete blood count (CBC), as well as blood drawn prior to the antigen challenge and at Time = 120 min. Laboratory assessments conducted at Visit 3 included CBC as well as blood drawn prior to the antigen challenge and at Time = 120 min.

The investigator could provide 50 mg of Benadryl (diphenhydramine) as a rescue medication to the subjects if necessary. Barring or excepting this rescue medication, no treatments related to nasal obstruction or allergy were permitted while the subject was participating in the study.

### Study treatment

The study products were chewable active or placebo tablets. The active tablet contained a proprietary blend made of*C. coturnix* quail eggs (PBQE) and the matched placebo tablets contained sorbitol. Both active (SniZtop) and placebo tablets were provided by the study sponsor (Stragen Pharma SA, Geneva, Switzerland). The PBQE is manufactured according to quality standards required for dietary supplements and is controlled, including the content in proteins and ovomucoids, in order to ensure the final quality of the product.

Subjects were instructed to slowly chew two tablets after being administered the allergenic challenge. The enrolled subjects were assigned a randomization number to determine the sequence of study product administration. According to the blind crossover design, the subjects either received two tablets of the active compound concomitant to the allergenic challenge at Visit 2 and two tablets of the placebo at Visit 3 or vice versa, minimizing the risk of an order effect.

### Procedures

#### Antigen exposure protocol

The nasal allergen challenge was chosen to reproduce allergic rhinitis symptoms as studied previously by Scadding et al. (2012). At each visit, subjects were administered an allergenic challenge consisting of two sprays in each nostril of a standardized dose of 10,000 BAU/mL (BAU, bioequivalent allergy unit) of a combination of the following aerosolized antigens, globally designated as allergens (supplier: HollisterStier Allergy, Spokane, WA): tree pollen (mix of*Quercus rubra*,*Quercus virginiana*,*Quercus alba*; mix of*Betula papyrifera*,*Betula nigra*,*Betula alba*;*Juniperus ashei*;*Carya pecan* [*illinoensis*]); grass pollen (*Cynodon dactylon*,*Poa pratensis*,*Festuca elatior* [*pratensis*],*Sorghum halepense*,*Lolium perenne*); dust mites, cat dander, and dog dander. In our study, the dose of allergen was set at 10,000 BAU/mL based on the Scadding et al. study in which this allergen dose led to a substantial drop in average PNIF.

#### Peak nasal inspiratory flow

The PNIF meter was introduced in 1980 by Youlten. It consists of a face mask applied over the subject's nose. When the subject inhales while keeping the mouth closed, a cursor indicates the associated nasal inspiratory flow. PNIF is a recognized objective, reproducible measure of nasal patency (Ottaviano et al. 2006). Ottaviano et al. (2006) reported mean PNIF values of about 120–140 L/min among healthy subjects ranging from 16 to 84 years old. In our study, the objective assessment of nasal symptoms and response to allergen was performed at each visit through PNIF measurements using a PNIF meter consisting of a plastic face mask connected to a plastic tube. The mask was shaped to be comfortable on the face and was similar to a breathing mask applied to the face during anesthesia. Subjects were asked to exhale, then put the face mask over their nose and mouth, and then close the mouth and inhale sharply through the nose. The mask was then taken off of the face and the dial on the tube was measured, quantifying the movement that occurred during inhalation. The entire procedure lasted less than 30 sec. Subjects had their PNIF measured preallergen exposure and then at multiple time points postallergen challenge. As PNIF values appear to be related to procedure practice (Ottaviano et al. 2006), the subjects were asked to first perform two training tests with the PNIF device prior to allergen exposure. After allergen exposure, three consecutive PNIF measurements were performed and recorded at each time point; the recorded value at a given time point corresponds to the mean value of these three measurements. Of note, before beginning any assessments, subjects had to be acclimated in the room for 1 h, ensuring a washout from environmental allergens.

#### Rhinitis symptoms self-assessment

At each visit, at baseline, and after allergen challenge, nasal and ocular symptoms were assessed by each subject who rated their symptoms on a VAS. The symptoms included itchy nose, itchy eyes, runny nose, nasal congestion, watery eyes, and sneezing and were rated on a 100-mm VAS with 0 being*none* and 100 being*extreme*. VAS assessment is recognized to be a pertinent method for severity assessment of rhinitis (Bousquet et al. 2007).

### Statistical analysis

The sample size was calculated based on the main parameter, PNIF, using the result of an exploratory study performed on seven completed subjects. The criteria taken into account to estimate the number of subject was the mean value calculated over 120 min after administration of allergen on the observed improvement of PNIF. Based on this analysis, 34 pairs of subjects were needed with*α *= 0.05 and*β *= 0.2 (i.e., a 80% power). Considering a dropout rate of ˜20%, the study was designed to have an enrollment of 40 evaluable subjects (*n* = 40).

PNIF and VAS nasal symptom score were analyzed using generalized linear models. For the overall analysis, the factors considered were the subject, the product, the visit, and the time points as well as relevant factor interactions. A post hoc analysis (adjusted*t*-test on difference) was performed using the least square means and the probability estimated on time × product interaction differences. For the analysis time by time, the factors considered were the subject, the product, and the visit.

In addition, a Friedman test (nonparametric approach) was performed on PNIF to confirm the coherency of the results through both parametric and nonparametric approaches.

Hypothesis testing for each of the efficacy endpoints under investigation was tested with a Type I error*α* = 0.05.

## Results

### Population

Ninety-four healthy subjects were screened, of which 46 failed screening and 48 were enrolled and randomly assigned treatment. Of these 48 subjects, 43 completed the study, whereas five subjects attended only the first two visits and were then lost of follow-up. Mean age was 32 years old ranging from 20 to 58; 52% of subjects were males and 48% were females. Marital status and ethnicity were also recorded for each participant. The main reasons for nonqualification were related to selection criteria. The reasons for early termination included lost to follow-up (*n* = 4) and study termination on subject's request without safety issue (*n* = 1). Two subjects were considered borderline regarding inclusion criteria, one with BMI = 35.39 and one with baseline PNIF = 93.33 L/min; these subjects were nevertheless included after individual review by the investigator and were considered in the present analysis.

A per protocol (PP) analysis was performed for assessment of the efficacy parameters of the study. All subjects complying with protocol requirements (*n* = 43), that is, all volunteers except those with “early termination status,” were included in the analysis.

A PP subset omitting subjects 1077, 1083, 1065, and 1066 who exhibited extreme behavior was also constructed. The investigator identified those subjects in his review as being associated with values which were not consistent with the expected direction of change, even though they met all criteria for enrollment. This second dataset is of*n* = 39.

### PNIF outcome

PNIF was used to assess nasal patency related to inflammatory and obstructive causes. Three measures were taken at each time point and the mean of the three values was analyzed.

**Figure 2:**
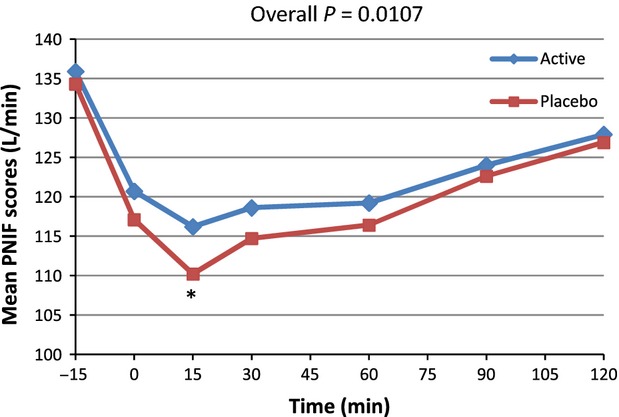
Average peak nasal inspiratory flow (PNIF) for active and placebo groups according to time in minutes for*n* = 43 (per protocol population). *Marginal significance at time 15 min,*P* = 0.0620 (post hoc analysis).

The PP analysis (*n* = 43) demonstrated that both the active and the placebo groups had sharp drops in PNIF values related to acute allergenic challenge, followed by a gradual increase in PNIF from nadir up to Time 120 reflecting the normal, gradual recovery from nasal obstruction induced by allergenic challenge. At all postchallenge time points, the active group had greater PNIF values compared to the placebo group indicating that the active product was associated with fewer symptoms and reduced intensity of these symptoms. The active and placebo group had the greatest difference in PNIF values at Time 15 (116.16 vs. 110.16, respectively) postchallenge demonstrating the rapid response related to administration of the active product, with immediate attenuation of the allergen-induced symptoms (Fig.2 and Table1). In both groups, a return to baseline PNIF values was progressively achieved over the following 120 min.

**Table 1:** Peak nasal inspiratory flow (PNIF) – comparison between active and placebo.

PNIF	Product	*N*	Mean (L/min)	SD (L/min)	SEM (L/min)
Preallergen exposure	Active	43	135.93	44.189	6.739
	Placebo	43	134.26	38.523	5.875
Time 0	Active	43	120.74	38.275	5.837
	Placebo	43	117.13	36.237	5.526
Time 15	Active	43	116.16	36.311	5.537
	Placebo	43	110.16	36.592	5.580
Time 30	Active	43	118.57	41.440	6.320
	Placebo	43	114.65	41.913	6.392
Time 60	Active	43	119.19	36.040	5.496
	Placebo	43	116.40	42.559	6.490
Time 90	Active	43	123.95	36.308	5.537
	Placebo	43	122.64	42.883	6.540
Time 120	Active	42	127.86	40.100	6.188
	Placebo	42	126.90	41.17	6.355

*N* represents the number of subjects having complete pairs for the time points in comparison. Time is expressed in minutes postallergen exposure.

**Figure 3:**
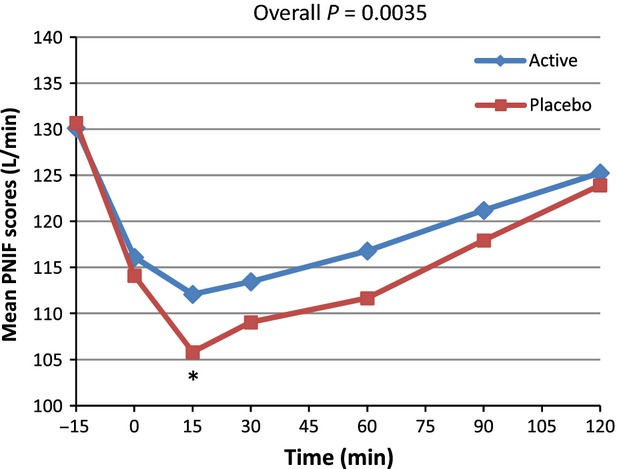
Average peak nasal inspiratory flow (PNIF) for active and placebo groups according to time in minutes for*n* = 39 (per protocol subset). *Marginal significance at time 15 min,*P* = 0.0556 (post hoc analysis).

From the PP subset (*n* = 39), similar results were observed, with the greatest difference in PNIF values between the active and placebo group observed at Time 15 (112.09 vs. 105.81) (Fig.3).

**Figure 4:**
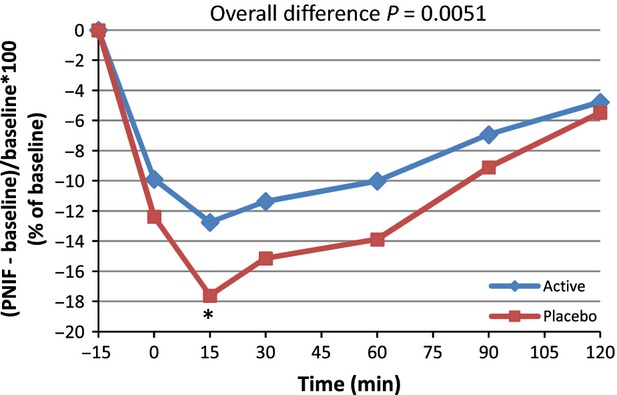
Average peak nasal inspiratory flow (PNIF) variation from baseline: (PNIF(t) − PNIF (pre)/PNIF (Pre) × 100) for active and placebo groups according to time in minutes for*n* = 43 (per protocol population). *Marginal significance at time 15 min,*P* = 0.0602 (post hoc analysis).

**Figure 5:**
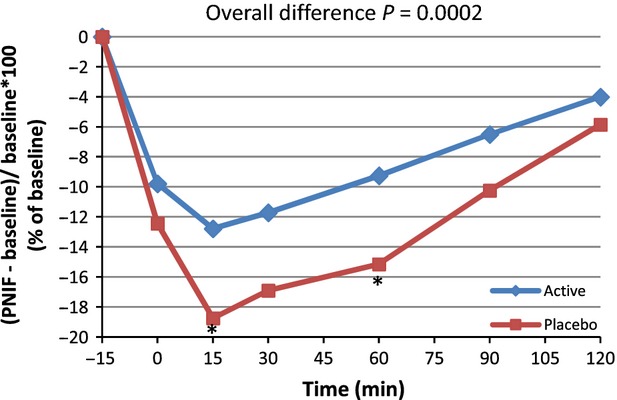
Average peak nasal inspiratory flow (PNIF) variation from baseline: (PNIF(t) − PNIF (Pre)/PNIF (Pre) × 100) for active and placebo groups according to time in minutes for*n* = 39 (per protocol subset). *Significant difference at time 15 and 60 min,*P* = 0.0271 and 0.0287, respectively.

From the PP analysis, a significant difference occurred in PNIF for the 0–120 min postchallenge period (*P = *0.0107). The overall means were 121.7 versus 117.6 for active and placebo, respectively. The multiple post hoc comparison showed a trend toward significance located at Time 15 min (*P = *0.0620). The nonparametric approach resulted in a significant difference for the 0–120 min time period (*P = *0.0410). When considering the data time by time, a significant difference occurred at 15 min (*P = *0.0235). A secondary analysis was performed on the variation versus baseline (the progression vs. baseline corrected parameters) for all points (PNIF(t) − PNIF (Pre))/PNIF (Pre) × 100 (Table2 and Fig.4). On the progression versus baseline corrected parameters, a significant overall difference was observed (*P = *0.0051) with a trend toward significance at Time 15 min (*P = *0.0602). Nonparametric analysis was performed and showed similar results. As presented in Figure4, when considering differences in PNIF value versus baseline expressed as a percentage of baseline, an improvement of 27.7% was observed at 15 min postchallenge in subjects taking the active product versus placebo, and an improvement of 23.8% for the active product at Time 90 versus placebo. Similar analyses were performed with the PP subset of*n* = 39. For PNIF values, consumption of the active product resulted in significantly improved breathing through the nose compared to placebo (*P =* 0.0035). The inference post hoc comparison shows a trend toward significance at Time 15 min (*P = *0.0556). When considering the data time by time, a significant difference occurred at 15 min (*P = *0.0290). Nonparametric analysis showed similar results. On the progression versus baseline corrected parameters an overall difference was observed (*P = *0.0002) with statistically significant differences at Time 15 and 60 min (*P = *0.0271 and 0.0287, respectively) and a trend toward significance at Time 30 (*P *= 0.0533). When considering the data time by time, the difference was mainly located at 15 min (*P =* 0.0261) and 60 min (*P *= 0.0582). Nonparametric analysis was performed and showed similar results. (Table2 and Fig.5). When considering differences in PNIF value versus baseline expressed as a percent of baseline, at Time 15 min postchallenge, administration of the active product resulted in an improvement of 31.8% compared to placebo. There was also an improvement of 36.5% for the active product versus placebo at Time 90.

**Table 2:** Peak nasal inspiratory flow (PNIF) – comparison between active and placebo on variation versus baseline.

(PNIF − baseline)/baseline × 100	Product	*N*	Mean (% of baseline)	SD (% of baseline)	SEM (% of baseline)
Preallergen exposure	Active	39	0.00	0.00	0.00
	Placebo	39	0.00	0.00	0.00
Time 0	Active	39	−9.79	13.03	2.09
	Placebo	39	−12.44	12.03	1.93
Time 15	Active	39	−12.79	17.19	2.75
	Placebo	39	−18.75	13.02	2.08
Time 30	Active	39	−11.72	20.06	3.21
	Placebo	39	−16.91	13.72	2.20
Time 60	Active	39	−9.26	20.19	3.23
	Placebo	39	−15.15	14.20	2.27
Time 90	Active	39	−6.51	16.45	2.63
	Placebo	39	−10.25	14.90	2.39
Time 120	Active	39	−4.01	14.96	2.40
	Placebo	39	−5.85	16.41	2.63
Preallergen exposure	Active	43	0.00	0.00	0.00
	Placebo	43	0.00	0.00	0.00
Time 0	Active	43	−9.89	13.26	2.02
	Placebo	43	−12.37	12.06	1.84
Time 15	Active	43	−12.75	17.75	2.71
	Placebo	43	−17.62	14.05	2.14
Time 30	Active	43	−11.36	20.19	3.08
	Placebo	43	−15.14	14.46	2.20
Time 60	Active	43	−10.01	20.72	3.16
	Placebo	43	−13.89	14.72	2.24
Time 90	Active	43	−6.93	18.30	2.79
	Placebo	43	−9.10	15.77	2.40
Time 120	Active	43	−4.78	16.51	2.52
	Placebo	43	−5.47	17.51	2.67

*N* represents the number of subjects having complete pairs for the time points in comparison. Time is expressed in minutes postallergen exposure.

### VAS outcome

**Figure 6:**
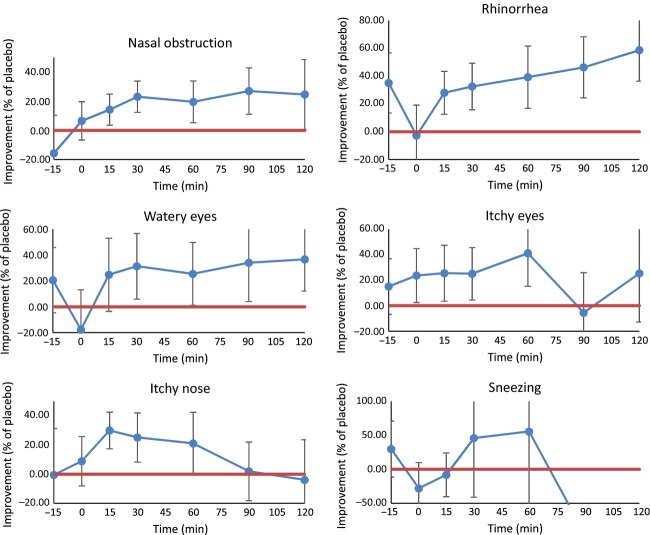
Percentage of improvement of average VAS results associated with active product versus placebo: (VAS_placebo_ − VAS_active_)/VAS_placebo_. All data above the red line indicates that the differences between the active product and the placebo are significant.

Consumption of the active product resulted in significant improvements versus placebo in subjects' perceived feelings of well-being based on subjective VAS scores for the following allergy-related symptoms: nasal obstruction (*P* = 0.0002), rhinorrhea (*P* = 0.0001), watery eyes (*P* = 0.029), itchy eyes (*P* = 0.006), and itchy nose (*P* = 0.009). No significant differences were found for sneezing (*P* = 0.5340) (Table3 and Fig.6).

**Table 3:** VAS symptoms – comparison between active and placebo.

VAS symptoms from 0 to 120 min	Product	*N*	Mean (no unit)	SD (no unit)	SEM (no unit)	*P*
Nasal obstruction	Active	41	17.07	16.18	2.527	0.0002
	Placebo	41	20.78	19.61	3.063	
Rhinorrhea	Active	41	8.92	14.61	2.282	0.0001
	Placebo	41	12.63	19.28	3.011	
Watery eyes	Active	41	7.13	14.15	2.210	0.029
	Placebo	41	8.9	18.7	2.920	
Itchy eyes	Active	41	7.08	13.97	2.182	0.006
	Placebo	41	9.34	18.21	2.844	
Itchy nose	Active	41	10.94	16.38	2.558	0.009
	Placebo	41	13.2	19.3	3.014	
Sneezing	Active	41	2.91	7.3	1.140	0.5340
	Placebo	41	2.62	7.91	1.235	

*N* represents the number of subjects having complete pairs for the time points in comparison.

### Total IgE outcome

No statistically significant difference was found regarding total IgE count between active and placebo groups both at preexposure (*P* = 0.272) and at Time 120 min (*P* = 0.250) (Table4). Based on these results, the active product did not appear to impact IgE antibody levels.

**Table 4:** Serum IgE – comparison between active and placebo.

SERUM IgE	Product	*N*	Mean (IU/mL)	SD (IU/mL)	SEM (IU/mL)	*P*
Preallergen exposure	Active	25	224.44	465.791	93.158	0.272
	Placebo	22	305.36	487.503	103.936	
Postallergen exposure	Active	25	224.68	464.643	92.929	0.250
	Placebo	22	307.14	490.131	104.496	

Sample size deviation was due to attrition and missing data. Significance testing was performed using Wilcoxon Mann–Whitney test.*N* represents the number of subjects. IU, International Unit.

### Rescue medication

No antiallergic rescue medication was required during the course of the study.

### Tolerance

The product was safe and well tolerated on the basis of a single administration of two tablets per visit. No serious or nonserious adverse events occurred during the study. Vital signs (blood pressure, heart rate, body temperature, respiratory rate) were assessed at each visit before the allergenic challenge and product administration and CBC was assessed prechallenge and at Time 120. No clinically significant changes were observed either for vital signs or for laboratory data in both groups.

## Discussion

In the present study, a standardized allergen challenge was used to induce allergic rhinitis symptoms in healthy subjects in order to determine the efficacy and safety of the dietary supplement, PBQE, on attenuating these symptoms. Allergic rhinitis expresses at the level of the nasal mucosa and eyes due to a transient immune reaction mediated by IgE antibodies. The corresponding episodic symptoms mainly include rhinorrhea, nasal obstruction, watery and itchy eyes, and sneezing (Pawankar et al. 2012).

This clinical trial was designed to employ a highly concentrated nasal allergen challenge consisting of a mixture of the most common outdoor and indoor allergens. The active study product provided significant improvements in PNIF score and associated allergy symptoms starting at Time 15 min postconsumption, which illustrates its fast-acting allergy-related benefits. Of note, improvements in the objective PNIF measurements of nasal obstruction correlate with the subjects' self-assessed perception of symptom relief. Parametric and nonparametric analyses showed similar results which strengthen the observed efficacy results of the active study product.

In this study, the active product was shown to be effective in healthy subjects overexposed to allergens. Under normal environmental conditions where occasional allergy sufferers are exposed to allergens, episodic allergy-related symptoms can be burdensome. When symptoms persist under these conditions, the repeated use of the active study product may provide long-lasting relief. This was demonstrated in previous double-blind, placebo-controlled clinical trials conducted by Bruttmann (1995). In these studies, subjects who were exposed to naturally occurring, continuous allergen challenges suffered from resulting perennial and pollen-induced seasonal rhinitis. Administration of quail egg homogenate to these subjects on a daily basis for a sustained period (several months in duration) resulted in significant improvements in allergy-related symptoms and a reduction in the use of rescue medications.

The results of this study showed that no modification of total plasma IgE occurred after consumption of the active study product. This reinforces the hypothesis that ovomucoid contained in the active product acts through a mechanism independent of IgE antibodies and, instead, attenuates the cascade of immune-related responses that results in an allergic reaction. This is consistent with the hypothesis that quail eggs ovomucoids inhibit serine proteases that act as chemical messengers involved in the allergic response. Further studies will be done to characterize the inhibitory action of the PBQE against specific serine proteases that are found in dust mites and pollen grains.

From shortly after the time of consumption following allergen exposure, the active study product provides significant attenuation of the allergic reaction and quick relief from allergy-related symptoms. This naturally derived product is devoid of major drawbacks and adverse effects such as drowsiness. However, the active study product should be contraindicated for subjects with a history of egg allergies.

## Conclusion

The dietary supplement consisting of PBQE was proven to be effective in attenuating allergy-related symptoms after exposure to a standardized allergenic challenge. Administration of an acute oral dose resulted in objective improvements in nasal breathing evidenced by PNIF measurements, as well as improvements of other allergy-related symptoms including itchy nose, itchy eyes, runny nose, nasal congestion, and watery eyes self-assessed by subjects' perceived feelings of well-being using subjective VAS Symptom Scores. Improvements in allergy-related symptoms were obtained 15 min after product intake, which demonstrates this product's fast-acting properties. The active study product was confirmed to be safe and tolerable, without the occurrence of adverse events during the study.
